# Identification of gene expression predictors of occupational benzene exposure

**DOI:** 10.1371/journal.pone.0205427

**Published:** 2018-10-09

**Authors:** Courtney Schiffman, Cliona M. McHale, Alan E. Hubbard, Luoping Zhang, Reuben Thomas, Roel Vermeulen, Guilan Li, Min Shen, Stephen M. Rappaport, Songnian Yin, Qing Lan, Martyn T. Smith, Nathaniel Rothman

**Affiliations:** 1 School of Public Health, University of California, Berkeley, California, United States of America; 2 Institute of Risk assessment Sciences, Utrecht University, Utrecht, the Netherlands; 3 Institute of Occupational Health and Poison Control, Chinese Center for Disease Control and Prevention, Beijing, China; 4 Occupational and Environmental Epidemiology Branch, Division of Cancer Epidemiology and Genetics, NCI, NIH, DHHS, Bethesda, Maryland, United States of America; University of Pittsburgh Graduate School of Public Health, UNITED STATES

## Abstract

**Background:**

Previously, using microarrays and mRNA-Sequencing (mRNA-Seq) we found that occupational exposure to a range of benzene levels perturbed gene expression in peripheral blood mononuclear cells.

**Objectives:**

In the current study, we sought to identify gene expression biomarkers predictive of benzene exposure below 1 part per million (ppm), the occupational standard in the U.S.

**Methods:**

First, we used the nCounter platform to validate altered expression of 30 genes in 33 unexposed controls and 57 subjects exposed to benzene (<1 to ≥5 ppm). Second, we used SuperLearner (SL) to identify a minimal number of genes for which altered expression could predict <1 ppm benzene exposure, in 44 subjects with a mean air benzene level of 0.55±0.248 ppm (minimum 0.203ppm).

**Results:**

nCounter and microarray expression levels were highly correlated (coefficients >0.7, *p*<0.05) for 26 microarray-selected genes. nCounter and mRNA-Seq levels were poorly correlated for 4 mRNA-Seq-selected genes. Using negative binomial regression with adjustment for covariates and multiple testing, we confirmed differential expression of 23 microarray-selected genes in the entire benzene-exposed group, and 27 genes in the <1 ppm-exposed subgroup, compared with the control group. Using SL, we identified 3 pairs of genes that could predict <1 ppm benzene exposure with cross-validated AUC estimates >0.9 (*p*<0.0001) and were not predictive of other exposures (nickel, arsenic, smoking, stress). The predictive gene pairs are *PRG2/CLEC5A*, *NFKBI/CLEC5A*, and *ACSL1/CLEC5A*. They play roles in innate immunity and inflammatory responses.

**Conclusions:**

Using nCounter and SL, we validated the altered expression of multiple mRNAs by benzene and identified gene pairs predictive of exposure to benzene at levels below the US occupational standard of 1ppm.

## Introduction

Benzene is a major industrial chemical and an extensive environmental contaminant present in traffic exhaust and cigarette smoke [1, 2]. It induces myelodysplastic syndrome and acute myeloid leukemia [3] and probably causes non-Hodgkin lymphoma [4] and other hematopoietic neoplasms [5–8]. In the U.S., occupational exposure levels are typically below 1 part per million (ppm) [9], the current permissible occupational exposure limit [10]. Development of biomarkers of exposure to benzene, particularly in people exposed below 1 ppm, would be a useful step towards improving risk assessment and minimizing adverse health effects.

We previously conducted a cross-sectional molecular epidemiological study of benzene exposure in factory workers in China, in which we found decreased white blood cell (WBC) counts in workers occupationally exposed to < 1 ppm benzene compared with non-occupationally exposed controls, and a highly significant dose-response relationship [11] with no apparent threshold within the occupational exposure range (0.2 to 75 ppm benzene) [12]. Other groups have reported effects at low levels of benzene, including increased blood mitochondrial DNA copy number and altered global and gene-specific DNA methylation [13], increased micronuclei [14], reduced expression of CD80 and CD86 in monocytes, and increased levels of IL-8, suggestive of compromised adaptive immunity and immunosurveillance [15].

Previously, we sought to identify transcriptomic biomarkers of benzene exposure in peripheral blood mononuclear cells (PBMC) [16, 17], as these cells are accessible and altered gene expression reflects effects throughout the body [18, 19]. Through microarray analysis of 125 subjects, we reported that occupational exposure to benzene at a range of levels from <1 ppm to >10 ppm (n = 59), perturbed the expression of many genes and pathways compared to non-occupationally exposed controls [16]. We identified a 16-gene expression signature associated with all levels of benzene exposure [16] and later showed that differential expression of the majority of genes was not associated with PBMC cell composition [20]. Our large study incorporated precise, individual measurements of exposure, and accounted for multiple sources of biological and experimental variability. More recently, in a subset of 10 highly-exposed (>5 ppm) subjects and 10 control subjects matched by age, sex, and smoking status, we applied mRNA sequencing (mRNA-Seq) and confirmed some microarray genes and pathways and identified additional genes [17].

The goal of the current study was to identify a minimal number of genes for which altered expression could predict exposure to <1 ppm benzene. First, we used the nCounter platform from NanoString Technologies, which has advantages over microarrays and real-time PCR [21], to validate our previous transcriptomic findings. Being a digital count-based method, it is linear over a greater dynamic range than microarrays and has sensitivity similar to that of TaqMan real-time PCR. It has reduced technical variability and bias compared with RNA-Seq due to fewer processing steps such as mRNA enrichment. Finally, it can measure the expression of up to 800 transcripts simultaneously from 100 nanograms (ng) RNA. Second, we used SuperLearner [22], an innovative ensemble machine learning methodology, to identify gene expression biomarkers predictive of <1 ppm benzene exposure.

## Materials and methods

### Study subjects

The subjects were from a molecular epidemiology study of occupational exposure to benzene that comprised benzene-exposed shoe manufacturing workers and non-occupationally exposed age- and sex-matched clothes-manufacturing workers from factories in the same region near Tianjin, China [11, 23]. This study complied with all applicable requirements of the U.S.A. and Chinese regulations, including IRB approval. Participation was voluntary and written informed consent was obtained. Exposure assessment to benzene was performed as described previously [23].

Biologic sample collection was described previously [23, 24]. Field-stabilized samples were transported on dry ice and RNA was isolated by the mirVana^™^ miRNA isolation kit (Applied Biosystems, Austin, TX). All RNA samples analyzed had A_260_:A_280_ and A_260_:A_230_ ratios between 1.7 and 2.1, and had distinct 28S and 18S rRNA bands with approximately 2:1 ratios following denaturing agarose gel electrophoresis.

Previously, we analyzed gene expression by microarray in 42 control subjects and 83 subjects exposed to benzene exposure levels ranging from <1 ppm to > 10 ppm [16]. We had sufficient RNA material left from 90 subjects for analysis by nCounter. The 90 subjects include 33 controls, 44 subjects exposed to <1 ppm benzene, 9 subjects exposed to 5–10 ppm, and 4 subjects exposed to > 10 ppm. Twenty subjects, including 10 controls and 10 subjects exposed to ≥ 5 ppm, were also previously analyzed by mRNA-Seq [17]. Demographic and exposure details are provided in Table 1. The mean air benzene level in the 44 <1ppm subjects was 0.55±0.248 ppm and the minimum level was 0.203 ppm.

**Table 1:** Characteristics of study subjects.

Exposure Category	Subject (n)	Benzene (ppm)	WBC count (per μl blood)	Age (Years)	Gender		Current Smoking	
					Male	Female	Yes	No
Control	33	0.035	6261 (1642)	29 ± 8.6	14 (36.8)*	19 (36.5)	7 (41.2)	26 (35.6)
< 1 ppm**	44	0.55 *±* 0.248^#^	5466 (1271)	28.9 ± 8.8	22 (58)	22 (42.3)	8 (47.0)	6 (49.3)
5–10 ppm	9	6.98 *±* 1.1	5344 (1518)	27.9 ± 8.5	1 (2.6)	8 (15.4)	1 (5.9)	8 (11.0)
≥ 10 ppm	4	30.69 *±* 25.64	4700 (455)	29.3 ± 15.3	1 (2.6)	3 (5.8)	1 (5.9)	3 (4.10)

*Subject number (%),

**Average level of benzene <1 ppm (in the 3 months prior to phlebotomy),

^#^ mean air benzene level ± SD values. WBC, white blood cells

### Selection of genes

The nCounter probeset comprised genes previously detected as differentially expressed by microarray (26 genes in 125 subjects) [16] and by mRNA-Seq (4 genes in 20 subjects) [17], and 3 reference genes. Details including RefSeq IDs, FDR-adjusted *p*-values and benzene-induced fold changes in expression are provided in S1 Table. Differential expression of the microarray data was previously analyzed using linear mixed effects models to estimate the log fold change in expression for each gene between control subjects and categories of benzene exposure, including <1 ppm, 5–10 ppm, and >10 ppm [16], with adjustment for multiple testing by Benjamini-Hochberg [25]. Expression of most of the 26 microarray-selected genes was significantly increased across multiple exposure categories relative to the controls, with average ratios >1.5. Significant decreases in *CEBPA* expression occurred in 3 exposure categories (0.7-fold average ratio) and of *MPL* at >10 ppm only (0.45).

For the RNA-Seq data, negative binomial models were previously used to estimate fold changes in gene expression between exposed (>5 ppm) and control subjects, with adjustment for multiple testing by Benjamini-Hochberg (n = 184) and a chi-squared goodness of fit t-test (n = 146) [17]. Of the 4 genes selected from the RNA-Seq study, 3 were significantly upregulated (*CMYA5*, *TTC9B*, *PLCL1*) and 1 (*ZNF703*) was downregulated, ≥1.5-fold.

We selected 3 reference genes that were reported in the literature to be stably expressed in PBMCs: the widely-used *beta-2 microglobulin (B2M)*, as well as *ribosomal protein large*, *P0 (RPLP0)* and *phosphoglycerate kinase 1 (PGK1)* [26].

### nCounter assay

We used the nCounter platform from NanoString Technologies to analyze the expression of the 33 selected mRNAs in 100 ng RNA from the 90 study subjects. The automated platform uses two 50 base pair probes per mRNA that hybridize in solution: a Reporter Probe that carries a fluorescent molecule barcode and a Capture Probe that enables the complex to be immobilized for data collection [21, 27].

The specific mRNA regions targeted, NanoString probe IDs, and melting temperatures of the probe pairs are detailed in S2 Table. Six technical replicates were included to assess replication and account for batch effects. Six positive control probes (POS A-F) and their corresponding RNA targets at various concentrations from 128 fM to 0.5 fM were included in the assay to account for systematic variation introduced by pipetting, sample purification, and imaging. Negative control probes (with no corresponding targets, NEG A-H) were included to control for non-specific background noise, i.e. non-specific carryover of reporter probes. The 96 samples were distributed across 8 batches for processing (S3 Table). Raw target counts were collected using the NanoString data collection software, nSolver. The raw target counts were background corrected, normalized to the mean of the positive control probes for each assay, and then normalized to the geometric mean of the reference genes (*B2M*, *PGK1*, *and RPLP0*). Thus-normalized target gene counts were analyzed by unsupervised clustering in Multiple Experiment Viewer software [28], using default settings, after being log2-transformed and mean centered by gene (row).

### Correlation of expression levels across platforms

For all 33 genes, we measured the strength of the association between the log2 transformed microarray intensities and log2 transformed nCounter normalized counts in all 90 subjects, and between the RNA-Seq and nCounter normalized counts for the 19 subjects in common between the studies. Using the *cor*.*test* function in R and normalized data, we calculated Pearson and Spearman correlation coefficients and their corresponding *p*-values, the latter to test whether the coefficients were significantly different from zero. A benefit of the less parametric Spearman’s rank correlation coefficient is that it does not matter if the scales of the expression measures of the two platforms are equivalent or if they are linearly related as one can simply measure the monotonic association between them. However, both Pearson and Spearman approaches to calculating p-values assume that gene expression across all subjects, as measured on each platform, have independent, normal distributions.

### Differential expression analysis of nCounter data and comparison with microarray and RNA-Seq

Differential expression analysis of the nCounter data (after excluding a randomly selected replicate from each replicate pair) was done by negative binomial regression and an empirical Bayes method to moderate the gene-wise quasi-likelihood dispersions, using the *glmQLFit* function in the edgeR package (https://bioconductor.org/packages/release/bioc/html/edgeR.html). The estimated coefficients from the gene-wise models are used as estimates of log fold changes in expression due to three levels of benzene exposure (<1 ppm, 5–10 ppm, and >10 ppm). The *glmQLFTest* function uses the estimated dispersions to compute moderated F-statistics to test whether all the exposure level coefficients are equal to zero, versus having at least one coefficient different from zero. It is a moderated F-statistic because the *glmQLFTest* function shrinks the dispersions and then uses these to compute a slight variation on the typical F-statistic. The negative binomial regression analyses were done in two ways: (1) without adjusting for any variables other than the three binary benzene exposure level variables (<1 ppm, 5–10 ppm, >10 ppm), and (2) adjusting for benzene exposure, smoking status, age, batch, and gender.

### SuperLearner approach to identify mRNAs predictive of benzene exposure

Going beyond the usual differential expression analysis, we sought to build predictors of benzene exposure. Specifically, the goal was to build a function that could take as input the expression levels of the 30 non-reference genes (or a subset of them) for any given subject, and generate as output the estimated probability that the subject has been exposed to benzene. In mathematical notation, the goal is to build a predictor function E[Y|X] = P(Y = 1|X), where Y is the binary indicator of benzene exposure at the <1 ppm level and X is a vector of 30 or fewer gene expressions. We focused on < 1 ppm benzene because 1 ppm is the current U.S. occupational standard [10] and we had a sufficient sample size for this exposure level. Thus, 33 control subjects and 44 subjects exposed to < 1 ppm were included in the analysis. The mean air benzene level in the <1ppm group was 0.55 ± 0.248 ppm, and the minimum exposure level was 0.203 ppm. Before building the prediction functions, differential expression analysis of the nCounter data from this subset of 77 subjects was performed as described above, both with and without adjustment for smoking status, age, batch, and gender.

The SuperLearner (SL) algorithm [29] uses a cross-validation procedure to test a combination of a user-specified set of candidate prediction algorithms. It is available as a statistical package *CVSuperLearner* [30] in the programming language, R [31]. SL is based on the statistical theory, Oracle Inequality [22, 32], which posits that, under certain assumptions, using cross-validation to select the best performing algorithm is equivalent to the so-called Oracle Selector (choosing the algorithm by knowing the true model), even if a very large number of selectors are used. The ability to use a library of statistical modeling techniques from simple to more complex, offers gains over any specific candidate algorithm in terms of flexibility to accurately fit the data, and potentially more precise prediction.

We built an exposure prediction function using the *CVSuperLearner* package in R, specifying 10 folds for cross-validation and a library of the following 6 learners: logistic regression "SL.glm", stepwise regression with Aikake Information Criterion "SL.stepAIC", Bayesian generalized linear model [33] "SL.bayesglm", Random Forest [34] SL.randomForest", generalized additive models (with different levels of smoothing) [35] "SL.gam", and "SL.mean". ‘SL.mean’ takes the sample average of the outcomes in the training set as the predicted value of the outcomes in the left out fold, for each iteration of the internal cross-validation in SuperLearner. We included a variety of algorithms that relied on different models and assumptions (e.g. tree-based algorithms, linear algorithms, etc.), and that were appropriate for the binary nature of the outcome variable (benzene exposure). A library with six algorithms has the advantage of being computationally efficient.

The *CVSuperLearner* function split the 77 by 30 expression matrix into 10 folds, and for each fold, each of the 6 learners in the library was trained on all folds but that one fold, and tested on the fold left out. Thus, as a result each learner has a vector of cross-validated predicted probabilities of exposure, (Z_1_, …,Z_6_). *CVSuperLearner* then chooses the best convex combination of the proposed learners by running a logisitic regression of the exposure outcomes on the predicted probabilities from each learner, P(Y = 1|Z) = expit(β_1_Z_1_ + ⋯ +β_6_Z_6_), thereby earning its name as an ensemble method. The best convex combination is defined as the combination that minimizes the expected squared error loss function for this regression problem. The final prediction function is therefore expit(β_1_Z_1_ + ⋯ + β_6_Z_6_). Finally, the resulting prediction function was externally cross-validated to assess the performance of the final prediction function.

First, using all 30 non-reference genes and using the *cvAUC* package in R, we built a benzene exposure prediction function. An area under the receiver operating characteristic curve (AUC) estimate is equivalent to the Wilcoxon-Mann-Whitney statistic, and can be thought of as the probability that the fitted classification model will rank a randomly chosen benzene-exposed sample higher than a randomly chosen control sample [36]. Therefore, the higher the AUC statistic, the better the classifier is. Confidence intervals and P-values for the cross-validated AUC estimates were computed with the *ci*.*cvAUC* function which uses influence curves to estimate standard errors.

Next, we then sought to determine the least number of genes needed to build a predictor whose performance was within 5% of this original cross-validated AUC estimate, i.e. to identify a smaller subset of genes that would build a prediction function that is virtually just as accurate as if all 30 genes were used. A predictor with fewer genes would be more practical to measure as a biomarker. To this end, genes were added in a forward stepwise fashion to the SL function, one at a time, and each time the performance of the resulting prediction function was assessed using the same *cvAUC* function [36]. Thus, all 30 genes were used to build a prediction function one at a time, and the gene with the highest cross-validated AUC was kept. Then, the 29 remaining genes were added to it and a prediction function was built out of the 29 pairs, keeping the pair with the highest cross-validated AUC. This process was continued until enough genes were added to get within 5% of the original cross-validated AUC estimate when all of the 30 genes were used.

### Expression of the benzene-predictive mRNAs at low-level benzene exposure

We plotted the expression of the benzene-predictive mRNAs with continuous benzene exposure in the 33 control and 44 subjects exposed to <1 ppm benzene. As air benzene levels are below the level of detection in the control subjects, we estimated benzene exposure using unmetabolized urinary benzene levels as described previously [37]. We previously reported that urinary benzene and mean individual air levels of benzene were strongly correlated (Spearman *r* = 0.88, *P* < 0.0001) in the epidemiologic study population [11] and in the subgroup of subjects analyzed in our previous microarray gene expression study (Spearman *r* = 0.76, *P* < 0.0001)[16]. We fitted a generalized additive model (gam) smoothing curve to each plot, to show the general trend in gene expression with urinary benzene.

### Assessment of predictive biomarker exclusivity

We assessed the exclusivity of our identified gene predictors in predicting benzene exposure as opposed to other factors. First, we identified 6 human PBMC transcriptome studies with data available in the Gene Expression Omnibus (GEO) database [38] in which subjects were exposed to various chemicals/lifestyles: nickel exposure (GSE40392)[39], arsenic exposure (GSE57711)[40], smoking status (GSE12587)[41], psychosocial stress (GSE25837)[42]; or had an immune or inflammatory-related disease: rheumatoid arthritis (RA, GSE15573)[43], peripheral arterial disease (PAD, GSE27034)[44]. In each data set, the status of the factor of interest was binary (i.e. smoker vs. non-smoker, diagnosed with PAD vs. healthy, exposed to nickel occupationally vs. exposed to nickel environmentally, etc.). Second, for each data set, the *limma* [45] package in R was used to fit unadjusted linear models, using all of the genes in the study. No covariates were added to the regressions except for the binary exposure status. As before, the *eBayes* function in R was used to create moderated t-statistics to test whether the exposure coefficients were significantly different from zero, and p-values were adjusted for multiple testing using the Benjamini-Hochberg method. Third, the gene predictors of benzene exposure were tested on data from the six PBMC studies. Specifically, the CVSuperLearner function was used to build exposure prediction functions for each of the 6 studies, training the six prediction functions on the gene expression data from each of the six studies. The cvAUC function was used to assess the performance of each of these prediction functions, i.e. their ability to accurately predict their associated exposure/condition. Furthermore, as an alternative or additional measure of biomarker exclusivity, we used the prediction function which was trained on the benzene exposure data using CVSuperLearner to predict the six different exposures/conditions from the PBMC studies, and assessed prediction performance using estimated AUC values.

## Results

### Performance of nCounter data

Raw and normalized nCounter data for all control probes and genes are presented in S4 and S5 Tables. These data are also available in the Gene Expression Omnibus database [38], accession number GSE119533. Hybridization performance for the 96 samples is illustrated in S1 Fig, in which the squares of the Pearson Correlations (R^2^) of positive control RNA target concentration vs. counts are plotted. Expected correlation is R^2^>0.95 and the observed correlations are 0.98–1.0. In the inset, the 6 positive control probes (POS A-E) counts are plotted vs. RNA target concentration for one representative assay. POS_E detects a target RNA at 0.5 fM, equivalent to approximately 1 RNA copy per mammalian cell, when 100 ng RNA (10,000 cells) is hybridized in the assay. POS_E count is greater than the average negative control counts (background) in all assays.

nSolver computes a normalization factor based on the average of positive control counts for the whole dataset. POS control normalization factors in our data were close to 1 (recommended range 0.3–3.0), suggesting minimal variation in mRNA content between samples (S2 Fig). In the unsupervised clustering analysis, technical replicates clustered together regardless of cartridge position and there was a broad separation of controls and exposed subjects based on expression profile, rather than batch (S3 Fig). The 6 pairs of technical replicates each had a Pearson correlation coefficient >0.999.

### Correlation of expression levels between nCounter and microarray and RNA-Seq platforms

In the 90 subjects analyzed by both nCounter and microarray, there was a good correlation in expression levels for most genes (Table 2). Among the 26 microarray-selected genes, 24 genes had Pearson and Spearman Rank correlation coefficients > 0.7 and associated *p* values < 0.05, and 20 and 19 genes had Pearson and Spearman Rank correlation coefficients greater than 0.85, respectively. Of the 4 RNA-Seq-selected genes, microarray expression levels of 3 genes (*CMYA5*, *ZNF703*, *TTC9B*) were poorly correlated with the nCounter data, with coefficients below 0.5, while *PLCL1* was better correlated (Pearson, 0.547; Spearman, 0.634).

**Table 2:** Correlation of expression levels between microarray and nCounter in all 90 subjects.

Gene Symbol	Pearson		Spearman	
	Coefficient	*p*-value	Coefficient	*p*-value
*Microarray-selected*				
*SERPINB2*	0.994	0	0.994	3.94E-93
*IL6*	0.993	0	0.990	3.04E-81
*IL1A*	0.982	0	0.979	1.13E-66
*AQP9*	0.981	0	0.985	3.78E-73
*CCL20*	0.975	0	0.972	7.59E-61
*TNFAIP6*	0.968	0	0.959	4.76E-53
*CLEC5A*	0.964	0	0.962	1.04E-54
*PTX3*	0.956	0	0.947	3.17E-48
*DRAM1*	0.954	0	0.951	1.02E-49
*IFNB1*	0.951	0	0.946	8.07E-48
*NFKB1*	0.947	0	0.947	2.70E-48
*F3*	0.927	0	0.948	1.07E-48
*KCNJ2*	0.925	0	0.922	2.12E-40
*PTGS2*	0.921	0	0.903	2.88E-36
*IL1RN*	0.921	0	0.891	5.16E-34
*GPR132*	0.901	0	0.899	1.59E-35
*CEBPA*	0.889	0	0.903	3.02E-36
*MPL*	0.883	0	0.908	2.30E-37
*CD44*	0.873	0	0.842	5.58E-27
*PRG2*	0.850	0	0.899	1.70E-35
*PLAUR*	0.818	0	0.830	1.27E-25
*UPB1*	0.804	0	0.821	1.31E-24
*ACSL1*	0.788	0	0.808	2.72E-23
*SOD2*	0.757	0	0.749	1.66E-18
*SLC2A6*	0.551	6.00E-09	0.576	7.92E-10
*DNAAF1*	-0.112	2.77E-01	-0.077	4.58E-01
*RNA-Seq-selected*				
*PLCL1*	0.547	8.11E-09	0.634	3.93E-12
*TTC9B*	0.125	2.26E-01	0.089	3.88E-01
*ZNF703*	0.084	4.15E-01	0.036	7.25E-01
*CMYA5*	0.031	7.64E-01	0.140	1.74E-01
*Reference*				
*RPLP0*	0.557	3.76E-09	0.518	6.38E-08
*B2M*	0.434	9.89E-06	0.475	1.01E-06
*PGK1*	-0.110	0.28557324	-0.117	2.56E-01

In the 19 subjects analyzed by both RNA-Seq and nCounter, there was a poor correlation in expression levels for most genes (Table 3). Only 7 genes, all microarray-selected genes, had correlation coefficients greater than 0.7, by Pearson’s (*CEBPA*), Spearman’s (*TNFAIP6*, *CLEC5A*, *SERPINB2*) or both methods (*IL1A*, *CCL20*, *AQP9*).

**Table 3:** Correlation of expression levels between mRNA-Seq and nCounter in 19 subjects.

Gene Symbol	Pearson		Spearman	
	Coefficient	*p*-value	Coefficient	*p*-value
*Microarray-selected*				
*IL1A*	0.898	4.13E-07	0.869	2.80E-06
*CCL20*	0.893	6.33E-07	0.763	3.56E-04
*AQP9*	0.736	4.93E-04	0.746	5.66E-04
*CEBPA*	0.731	5.73E-04	0.647	4.61E-03
*KCNJ2*	0.683	1.77E-03	0.574	1.27E-02
*DRAM1*	0.659	2.96E-03	0.556	1.82E-02
*PTX3*	0.652	3.39E-03	0.653	4.13E-03
*TNFAIP6*	0.645	3.83E-03	0.713	8.85E-04
*NFKB1*	0.641	4.15E-03	0.467	5.22E-02
*GPR132*	0.621	5.96E-03	0.591	1.12E-02
*CLEC5A*	0.579	1.19E-02	0.933	1.58E-08
*IFNB1*	0.522	2.64E-02	0.503	3.54E-02
*MPL*	0.506	3.21E-02	0.642	4.07E-03
*ACSL1*	0.473	4.75E-02	0.587	1.19E-02
*PRG2*	0.461	5.41E-02	0.463	5.45E-02
*IL6*	0.407	9.38E-02	0.472	5.00E-02
*PTGS2*	0.363	1.39E-01	0.408	9.43E-02
*UPB1*	0.333	1.77E-01	0.389	1.11E-01
*SERPINB2*	0.325	1.89E-01	0.717	1.15E-03
*SOD2*	0.310	2.10E-01	0.393	1.07E-01
*IL1RN*	0.290	2.43E-01	0.329	1.82E-01
*SLC2A6*	0.158	5.31E-01	0.216	3.88E-01
*PLAUR*	0.096	7.04E-01	0.251	3.14E-01
*F3*	-0.007	9.77E-01	0.366	1.36E-01
*CD44*	-0.051	8.42E-01	-0.005	9.87E-01
*DNAAF1*	-0.073	7.74E-01	0.124	6.24E-01
*RNA-Seq-selected*				
*ZNF703*	-0.002	9.92E-01	0.019	9.41E-01
*PLCL1*	0.322	1.92E-01	0.267	2.82E-01
*TTC9B*	0.100	6.93E-01	0.209	4.03E-01
*CMYA5*	-0.267	2.83E-01	-0.278	2.64E-01
*Reference*				
*RPLP0*	0.544	1.95E-02	0.529	2.57E-02
*PGK1*	0.001	9.98E-01	-0.009	9.74E-01
*B2M*	0.160	5.27E-01	0.110	6.62E-01

### Comparison of nCounter and microarray differential expression in all 90 subjects

nCounter data in all 90 subjects was analyzed by negative binomial regression in control and benzene-exposed (<1 ppm, 5–10 ppm and >10 ppm) subjects, with adjustment for covariates. Among the 26 microarray-selected genes, 23 were confirmed as differentially expressed (FDR *p*-value <0.05) by nCounter, with 22 up-regulated and 1 (*CEBPA*) down-regulated (Table 4). Among the 3 non-validated genes, *DNAAF1* and *SLC2A6* expression level were poorly correlated between the two platforms (Table 2). Though *MPL* was highly correlated (Table 2), differential expression was not significant by nCounter. This is probably because differential expression in the microarray study was driven by the 13 subjects exposed to >10 ppm benzene, whereas only 4 of those subjects were analyzed by nCounter.

**Table 4:** Differential expression analysis of nCounter mRNAs in all 90 subjects.

Gene Symbol	Fold-change Exposed vs Controls			*p*-value	FDR
	<1 ppm	5–10 ppm	>10 ppm		
*Microarray-selected*					
*AQP9*	2.50	1.76	1.16	8.33E-13	2.75E-11
*DRAM1*	2.35	1.77	1.63	2.16E-11	3.56E-10
*IL1A*	2.79	2.41	2.13	6.40E-11	6.74E-10
*PTX3*	2.32	1.61	1.57	8.18E-11	6.74E-10
*KCNJ2*	2.30	1.84	1.61	5.77E-10	3.81E-09
*PTGS2*	1.91	1.63	1.11	1.32E-09	7.24E-09
*TNFAIP6*	2.69	1.71	1.56	2.79E-09	1.31E-08
*ACSL1*	1.91	1.50	1.10	3.40E-09	1.40E-08
*SERPINB2*	5.12	3.26	1.39	2.16E-08	7.90E-08
*F3*	3.58	2.41	1.12	3.87E-08	1.28E-07
*CD44*	1.85	1.62	1.33	5.09E-08	1.53E-07
*IFNB1*	3.41	2.37	1.38	2.65E-07	7.14E-07
*CCL20*	2.01	1.51	1.49	2.81E-07	7.14E-07
*CLEC5A*	2.48	1.88	1.14	7.89E-07	1.86E-06
*IL1RN*	1.72	1.56	1.07	5.42E-06	1.19E-05
*CEBPA*	0.55	0.63	0.67	7.79E-06	1.61E-05
*IL6*	3.21	2.73	1.49	9.61E-06	1.87E-05
*SOD2*	1.73	1.47	1.16	1.46E-05	2.67E-05
*NFKB1*	1.69	1.49	1.29	3.72E-05	6.13E-05
*PRG2*	1.90	1.50	1.35	1.06E-04	1.66E-04
*UPB1*	1.65	1.51	1.07	1.58E-04	2.36E-04
*GPR132*	1.59	1.41	1.43	3.95E-04	5.67E-04
*PLAUR*	1.40	1.39	0.82	5.55E-03	7.63E-03
*MPL*	0.77	0.65	0.72	6.81E-02	8.99E-02
*DNAAF1*	0.63	0.84	0.75	8.06E-02	1.02E-01
*SLC2A6*	1.20	1.03	1.04	4.60E-01	4.90E-01
*RNA-Seq-selected*					
*ZNF703*	0.55	0.61	0.57	1.88E-05	3.26E-05
*CMYA5*	0.80	0.84	0.90	3.83E-01	4.36E-01
*PLCL1*	0.86	0.88	1.18	4.36E-01	4.80E-01
*TTC9B*	0.90	0.90	1.02	8.68E-01	8.95E-01

FDR, false discovery rate

Among the 4 RNA-Seq-selected genes, ZNF703, which was significantly down-regulated by > 5 ppm benzene in the RNA-Seq data in 19 subjects (but not in the microarray data in 125 subjects), was significantly down-regulated in the nCounter data in 90 subjects. The remaining 3 RNA-Seq-selected genes were not differentially expressed in the 90 subjects by nCounter.

We did not do any analysis with the negative binomial model treating benzene exposure as an ordinal variable in the nCounter data given the small sample sizes for the 5–10 ppm and >10 ppm groups (9 and 4 respectively). For the same reasons, we feel we cannot draw any conclusions from Table 4 in terms of dose response. While the response seems to be consistently in the same direction for all levels of benzene exposure, it is hard to say more given the sample size.

### Comparison of nCounter and RNA-Seq differential expression in 19 subjects

nCounter data in 19 of the 20 subjects previously analyzed by RNA-Seq, was analyzed by negative binomial regression in control and benzene-exposed (5–10 ppm and >10 ppm) subjects, with and without adjustment for covariates. Differential expression of the 4 RNA-Seq-selected genes was not confirmed by nCounter in either the unadjusted (S6 Table) or adjusted (data not shown) models. Expression levels of these 4 genes were poorly correlated between the RNA-Seq and nCounter data (Table 3). In the unadjusted but not the adjusted model, several of the microarray-selected genes with good expression level correlation (Spearman coefficient >0.7) between RNA-Seq and nCounter, were found to be differentially expressed in the 19 subjects in the nCounter study.

### Identification of genes predictive of benzene exposure by SuperLearner

As most of our exposed subjects were exposed to <1 ppm benzene, we sought to identify gene subsets predictive of benzene exposure in these subjects. Analysis of the nCounter data for the 33 controls and 44 subjects exposed to <1 ppm, using the adjusted negative binomial model and controlling for multiple testing, revealed most genes as significantly differentially expressed (S7 Table). Using all 30 non-reference genes, *CVSuperLearner* built a benzene exposure (1 ppm) prediction function with a cross-validated AUC estimate of 0.96 (CI 0.89 − 1). Using an iterative SL approach to determine the least number of genes that could predict benzene exposure—run many times due to the randomness involved in choosing the folds for cross-validation—we found that each time only two genes were required to get a cross-validated AUC estimate >0.9 whereas a single gene alone could never build a prediction function with a cross-validated AUC estimate >0.9. Therefore, we determined that two genes were sufficient for building an accurate exposure predictor. All 6 Superlearner algorithms contributed to the prediction of benzene exposure across the gene pairs and no algorithms consistently contributed more than others.

Details of the 6 pairs of genes that were most frequently identified as accurate predictors of benzene exposure are listed in Table 5. All 6 genes in the biomarker pairs were significantly differentially expressed at the <1 ppm exposure level. The 2 pairs with the highest AUCs (0.94) were *IFNB1/NFKB1* and *PGR2/CLEC5A*. *ACSL1* and *CLEC5A* each featured in 3 pairs, *NFKB1* and *PRG2* in 2 pairs, and *IFNB1* and *AQP9* in 1 pair. The genes play roles in innate immunity response and energy homeostasis.

**Table 5:** Top 6 biomarker pairs predictive of benzene exposure identified by SuperLearner.

Biomarker Pair	AUC	CI	P-Value*
*AQP9*, *ACSL1*	0.91	0.82, 1	0
*NFKB1*, *IFNB1*	0.94	0.87, 1	0
*PRG2*, *ACSL1*	0.91	0.84, 0.99	0
*PRG2*, *CLEC5A*	0.94	0.86, 1	0
*NFKB1*, *CLEC5A*	0.92	0.84, 1	0
*ACSL1*, *CLEC5A*	0.91	0.83, 0.99	0

AUC, area under the curve; CI, confidence interval.

*P-values estimated to ten decimal points (one-sided test with null hypothesis of AUC = 0.5).

### Expression of the predictive mRNAs with continuous low-level benzene exposure

As shown in S4 Fig, for each benzene-predictive mRNA, expression of the controls (red dots) is distinct from that of the exposed group (blue dots) and the gam curves show an increase in expression from control to exposed status, with little dose-response apparent across urinary benzene levels in the < 1 ppm exposed subjects. We did not examine the expression of the predictor mRNAs at higher levels as they were selected based on data in the <1 ppm vs control group analysis. However, as shown in Table 4, the direction of the change in expression was the same for the benzene signature genes in the higher exposure groups as in the <1ppm group though the magnitude of the change in expression was more marked in the <1ppm group. Further, gene expression did not significantly differ between the 44 subjects exposed to <1 ppm and the 13 subjects exposed to >5 ppm subjects for any of the 30 genes analyzed (S8 Table). This suggests that the gene expression biomarkers identified may be predictive at any level of benzene exposure including < 1ppm but requires validation.

### Exclusivity of the biomarker pairs in predicting benzene exposure

We examined the ability of the 6 prominent gene pair predictors to predict other factors, including exposure/lifestyle (arsenic, smoking, nickel, psychosocial stress) and inflammatory disease (PAD, RA) in studies with available PBMC transcriptome data. Differential expression analysis of the respective datasets revealed that none of the 6 benzene predictor genes was altered by arsenic, PAD, RA, smoking and stress, compared with their respective controls, after adjusting for multiple testing. In the nickel study, only *NFKB1* was differentially expressed.

We assessed how well the benzene predictors could predict the selected non-benzene factors in two ways. First, we used the 6 gene pairs from our study to build new SL predictors for each outcome individually. As shown in S9 Table none of the 6 benzene predictor pairs were able to predict arsenic exposure or stress, based on low *cvAUC* estimates. The highest cross-validated AUC estimates for predicting smoking, RA and PAD were 0.69, 0.71 and 0.73, respectively. However, for nickel exposure, two biomarker pairs which include *NFKB1* (*NFKB1*, *IFNB1*) and (*NFKB1*, *CLEC5A*) had AUCs of 0.88. Second, we took the AUC values for each SL predictor pair fit onto the benzene data, i.e. the “benzene fit”, and used these to predict the other outcomes. As shown in S9 Table, most gene pairs were poor predictors. However, two pairs (*AQP9*, *ACSL1*) and (*NFKB1*, *IFNB1*) were predictive of nickel exposure, with AUCs >0.8 and p-values <0.05. Thus, considering the results of both methods of assessing predictivity, three benzene biomarker pairs are largely exclusive to benzene, particularly (*PRG2*, *CLEC5A*), and two biomarker pairs are also good biomarkers of nickel exposure.

## Discussion

In the current study, we validated our previous microarray findings in occupationally exposed subjects using the nCounter platform and used SuperLearner to refine pairs of genes whose expression could predict benzene exposure at low occupational levels (<1 ppm). Finding a small number of highly predictive genes could enable the development of sensitive gene expression assays, e.g. droplet digital PCR, that could be deployed inexpensively in large population studies in small quantities of blood.

nCounter Validation was performed on genes selected based on differential expression in our previous microarray and mRNA-Seq studies. The high correlation of nCounter and microarray data is in agreement with previously published studies [21, 46–48]. It is unclear why the nCounter data correlates poorly with the mRNA-Seq data in our study. Concordance in between RNA-Seq and nCounter data was reported in previous studies [49, 50].

As we sought to identify genes predictive of low-level benzene exposure, we first validated differential expression in the 44 subjects exposed to <1 ppm benzene in comparison with the 33 controls, using nCounter. We then used SL to find genes predictive of benzene exposure in this dataset. We chose SL as we did not want to rely on an incorrectly specified parametric model to build a predictor, as the resulting predictor would be biased, or to choose an arbitrary parametric model based on what gave the best prediction results. The SL algorithm allows the user to provide a variety of learners on a spectrum of data-adaptiveness or smoothness, and results in a less biased predictor with better inference [32]. Further, SL performs at least as well as the best single learner provided by the user, since cross validation is used to find the best weighted combination of the learners to build the final predictor. We selected the learners to avoid overfitting, especially given the small sample size, by including learners with different levels of data adaptiveness and smoothness that could be used with the binary exposure variable.

Previously, we used SL to estimate dose-related changes in the expression of pathway genes in response to benzene [20]. It is relatively novel to use SL to identify gene expression-based predictive biomarkers. SL was applied to identify gene expression predictors of metastasis in breast cancer and predictors of the presence of a cancerous tumor in prostate cancer [30] using publicly available microarray data sets [51, 52]. For breast cancer, SL attained a risk comparable to the best algorithm with a mean squared error of 0.194, and for prostate cancer, it outperformed even the best algorithm in the library with a mean squared error of 0.067.

In the current study, we found 6 pairs of genes (with overlapping members) that could discriminate the <1 ppm exposed and control groups. As differential expression analysis revealed no difference in expression between the low-level and high-level (>5 ppm) exposure groups, the gene pairs may be able to predict benzene exposure in levels ranging from <1 ppm to > 5 ppm but may not be able discriminate between low and high exposure levels.

We found that 3 gene pairs were highly predictive of low-level benzene exposure and were not strong predictors of smoking, RA, PAD, stress, arsenic, or nickel exposure. An additional 3 pairs were good predictors of both benzene and nickel exposure. A caveat to our approach is that we were only able to determine exclusivity by analyzing performance of our biomarker pairs in a limited number of available datasets with information on gene expression and exposure to a factor or disease of interest in human PBMC in GEO. Thus, we cannot exclude association of these biomarker pairs with other exposures. Based on currently available data, therefore, we have identified 3 pairs of gene expression biomarkers that are exclusively predictive of low-level occupational benzene exposure.

Strengths of our study are our cross-sectional study of occupational benzene exposure with well-characterized exposures, use of a cutting-edge digital counting method nCounter to validate benzene-induced differential gene expression, and the use of SL, an innovative data-adaptive approach to identify predictive biomarkers. Limitations of our study include the use of relatively few studies to determine exclusivity of biomarker prediction—due to a limited number of comparable publicly available studies—and a lack of validation of our identified biomarkers in an independent study population, something we hope to address in the future when we identify and gain access to a suitable, similarly well-characterized study population.

As mentioned in the introduction, several cellular and molecular markers of low-dose benzene exposure were identified previously [11–15]. Urinary benzene is a good biomarker for exposure to low levels of benzene [37, 53]. An advantage of gene expression markers is that they may inform biology and risk assessment. The genes in the pairs predictive of benzene exposure (*ACSL1*, *CLEC5A*, *NFKB1*, *PRG2*) play roles in innate immunity and inflammatory responses. *ACSL1* is induced in classically activated inflammatory macrophages and is causal to the enhanced inflammation and atherosclerosis associated with diabetes in mouse models [54, 55]. *CLEC5A* regulates inflammatory responses and activation of myeloid cells [56, 57]. It is expressed on human inflammatory macrophages in vivo [58] and is a critical receptor for some viruses [59–62], and bacteria [63] and mediates innate immunity inflammatory response. *CLEC5A* is also expressed on alveolar macrophages in mice exposed long-term to cigarette smoke (CS), as well as in human smokers, and it mediates macrophage function and chronic obstructive pulmonary disease pathology in mice [64]. NF-kappa-B is a pleiotropic transcription factor present in most cell types and is activated by various stimuli such as cytokines, oxidant-free radicals, ultraviolet irradiation, and bacterial or viral products. Activated NFKB stimulates the expression of genes involved in a wide variety of biological functions. Inappropriate activation of NFKB has been associated with a number of inflammatory diseases [65–67]. Polymorphisms in the *NFKB* gene may play a role in chronic lymphocytic leukemia development [68]. *PRG2* encodes an eosinphil cytotoxic secretory granule protein involved in innate immunity and immunopathology [69, 70].

It is not known why certain pairs of innate immunity / inflammatory response genes are predictive of benzene exposure over other pairs. Benzene metabolites have been shown to impact aspects of innate immunity [71–74]. Further, genetic polymorphisms in innate immunity genes may modify the risk of hematotoxicity in benzene-exposed workers [75]. However, future studies are needed to fully understand the effects of benzene exposure on innate immunity and the role of the gene pairs identified in the current study.

Nickel exposure is associated with nasal and lung cancer in nickel refinery workers [28, 76–78] and with contact hypersensitivity and dermatitis [79]. Alterations in innate and acquired immunity have been observed in animals [80]. The gene pairs predictive of both benzene and nickel exposure are *AQP9 / ACSL1* and *NFKB1 / IFNB1*. Nickel was previously known to activate NFKB signaling [81, 82]. AQP9 is an aquaglyceroporin channel transporter. It transports arsenic and mediates the cellular response to arsenic exposure [83–86]. AQP9 expression in neutrophils may play a role in establishing contact hypersitivity [87] and is enhanced in systemic inflammatory response syndrome [88]. It was reported to be one of a panel of five genes whose expression in PBMC could discriminate between patients with chronic inflammation and healthy controls [89]. IFNB1 is a cytokine member of the interferon family of signaling proteins. It is important for defense against viral infections and is involved in cell differentiation and anti-tumor defenses.

A limitation of PBMC gene expression markers is that they may reflect a short-term effect in currently-exposed individuals due to the short-lived nature of lymphocytes. However, changes in gene expression persisted in whole blood samples from former cigarette smokers up to 30 years after cessation compared with non-smokers, as reported in a recent meta-analysis [90], possibly through hypomethylation in the gene promoter regions [91]. Persistent effects on DNA methylation of multiple additional genes in former smokers has been reported [92–95], suggesting that it may be a more stable marker. We are exploring this cross-sectionally as we have blood samples from people currently and previously exposed to benzene enabling us to explore biomarkers of cumulative or persistent or long-term exposure cross-sectionally without the need for a prospective cohort study. Ultimately, a comprehensive exposure signature highly specific to benzene incorporating gene expression, DNA methylation, as well as other types of markers may be useful tool for risk assessment in the future.

## Conclusion

Using the nCounter platform, we validated the altered expression of 27 mRNAs in individuals occupationally exposed to <1 ppm benzene and identified 3 gene pairs that exclusively predict current benzene exposure. Our approach of using the cutting-edge digital counting method, nCounter, to validate differential gene expression, and of SL to identify predictive genes, has broad applicability in the field of environmental health. Future studies could explore whether the biomarkers are predictive of past benzene exposure, what roles (if any) they play in the toxicity and disease, and whether they can be modulated by factors such as diet to minimize risk.

## Supporting information

S1 Fig Linearity of the nCounter platform *vs*. RNA concentration. This graph demonstrates the linearity of the nCounter platform. The square of the Pearson Correlations (R2) of Positive control RNA target concentration vs. counts is plotted for all 96 samples. Inset: 6 POS control probes counts (y-axis) are plotted vs. RNA target concentration (x-axis) for one representative assay.
(PDF)

S2 Fig Positive control normalization factors. nSolver computes a normalization factor for each assay based on the average of positive control counts for the whole data set. NanoString recommends that for optimal results positive control normalization factors range between 0.3 and 3.0 for all assays. POS control normalization factors indicate minimal inter-assay technical variation.
(PDF)

S3 Fig Hierarchical clustering of nCounter gene expression. Normalized counts were analyzed by unsupervised clustering in MeV (Multiple Experiment Viewer) software, using default settings. Data were log2-transformed and mean centered by gene (row) prior to clustering. Replicates cluster together and there is a broad separation of control and exposed samples.
(JPG)

S4 Fig Expression of benzene predictor genes *vs*. continuous benzene exposure. For the control and <1ppm subjects and for each of the 6 genes, each subject’s urinary benzene level is plotted against their gene expression level. A GAM smoothing curve is fit using all subjects.
(JPG)

S1 Table Genes selected for inclusion in nCounter ProbeSet. (XLSX)

S2 Table Details of the nCounter ProbeSet. (XLSX)

S3 Table Randomization of samples across study variables. (XLSX)

S4 Table Raw nCounter data. (XLSX)

S5 Table Normalized nCounter data. (XLSX)

S6 Table Differential expression analysis of nCounter mRNAs for 30 genes in subjects previously analyzed by mRNA-Seq. (XLSX)

S7 Table Differential expression analysis of nCounter mRNAs in control and low-exposed subjects previously analyzed by mRNA-Seq. (XLSX)

S8 Table Differential expression analysis of nCounter mRNAs in low-exposed vs high-exposed subjects previously analyzed by microarray. (XLSX)

S9 Table Predictivity of gene pairs for benzene and other outcomes. (XLSX)
