# Supplementary figures and images for: Identification of gene expression predictors of occupational benzene exposure
Source: PLoS One. 2018 Oct 9;13(10):e0205427. doi: 10.1371/journal.pone.0205427 (PMC6177191; doi:10.1371/journal.pone.0205427)

## POS control Pearson correlation ( $R^2$ )

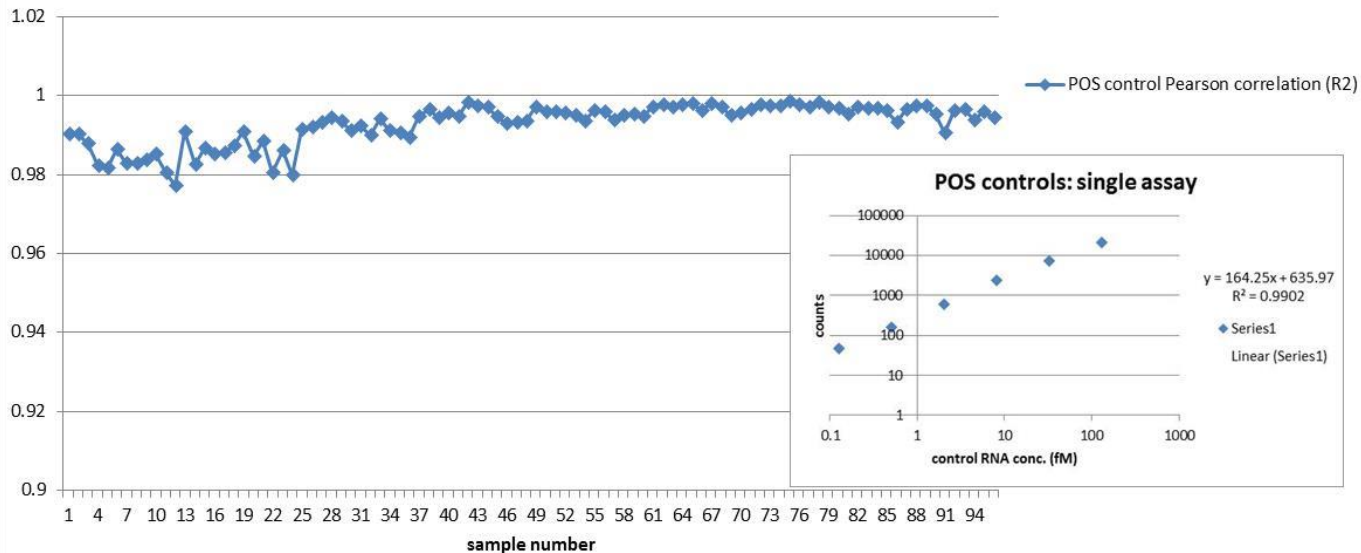

Supplement: S1 Fig — This graph demonstrates the linearity of the nCounter platform. The square of the Pearson Correlations (R2) of Positive control RNA target concentration vs. counts is plotted for all 96 samples. Inset: 6 POS control probes counts (y-axis) are plotted vs. RNA target concentration (x-axis) for one representative assay. (PDF) [file pone.0205427.s001.pdf]

## Assay variability: technical vs. biological

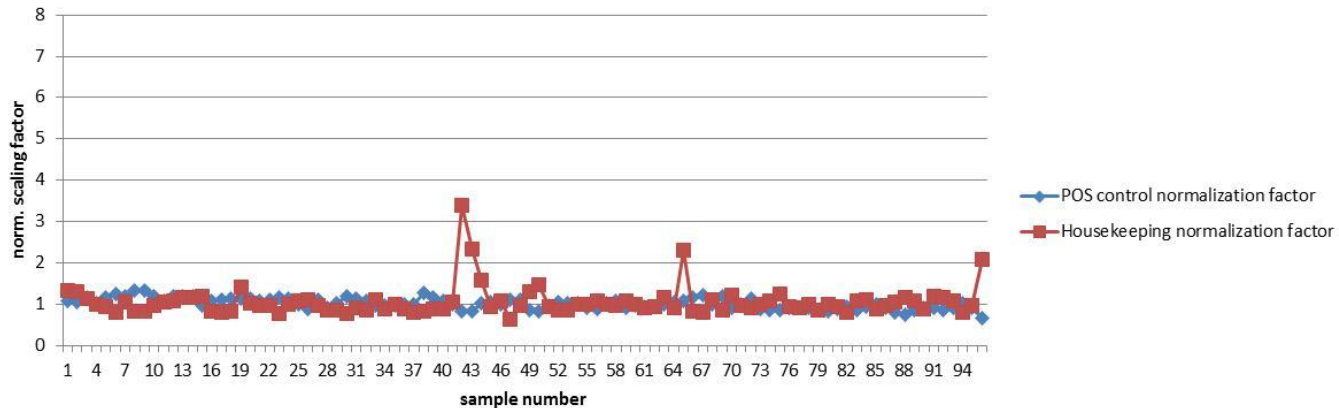

Supplement: S2 Fig — nSolver computes a normalization factor for each assay based on the average of positive control counts for the whole data set. NanoString recommends that for optimal results positive control normalization factors range between 0.3 and 3.0 for all assays. POS control normalization factors indicate minimal inter-assay technical variation. (PDF) [file pone.0205427.s002.pdf]

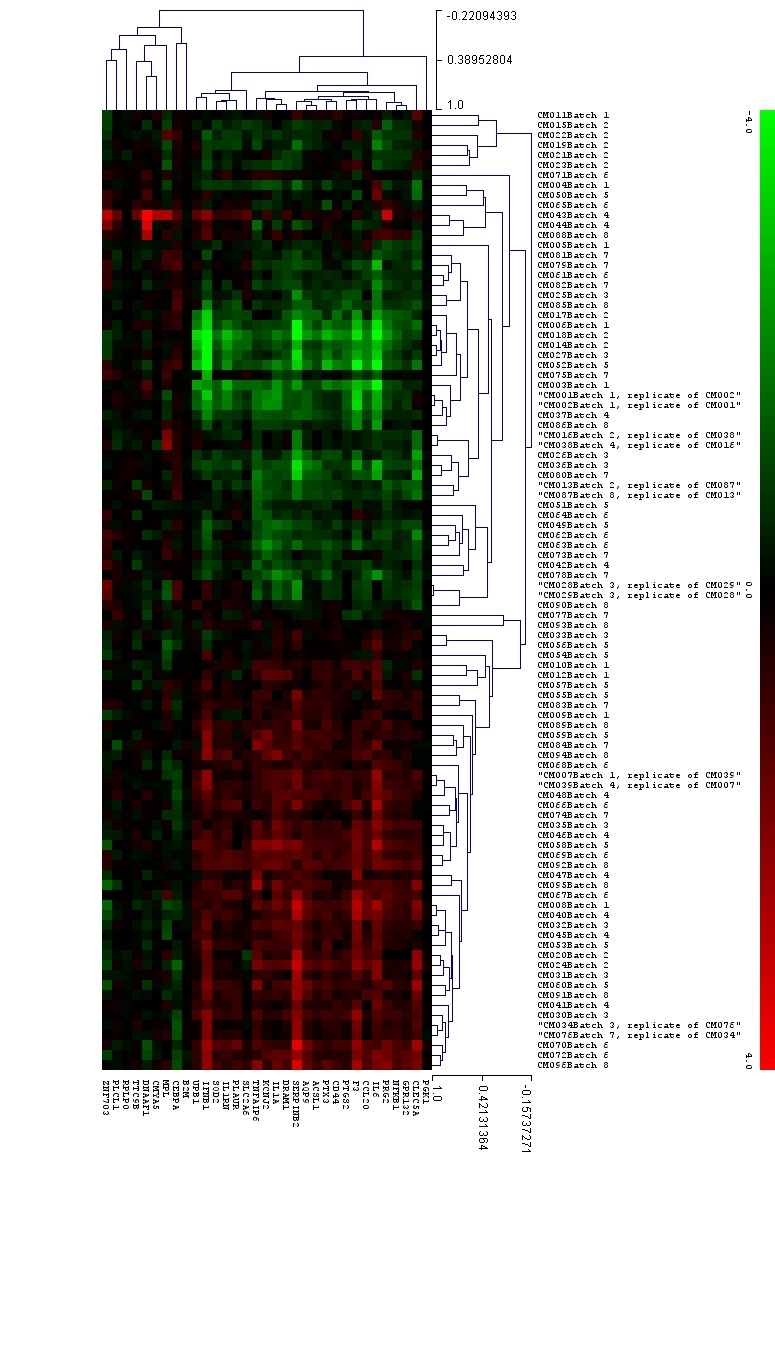

Supplement: S3 Fig — Normalized counts were analyzed by unsupervised clustering in MeV (Multiple Experiment Viewer) software, using default settings. Data were log2-transformed and mean centered by gene (row) prior to clustering. Replicates cluster together and there is a broad separation of control and exposed samples. (JPG) [file pone.0205427.s003.jpg]

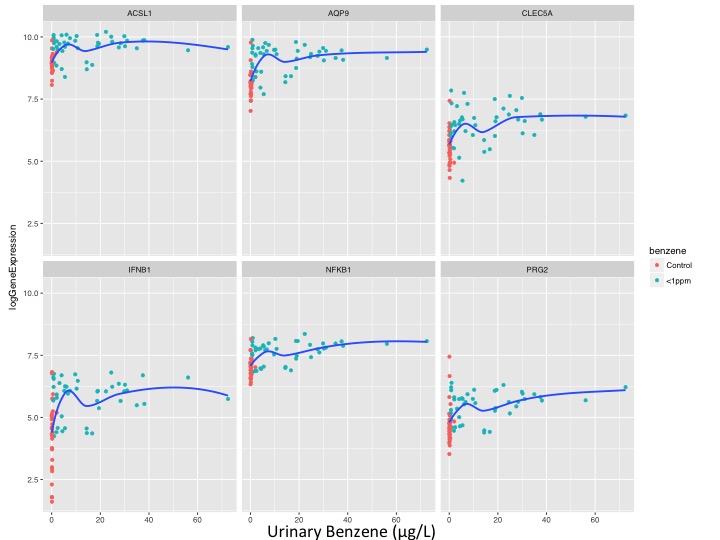

Supplement: S4 Fig — For the control and <1ppm subjects and for each of the 6 genes, each subject’s urinary benzene level is plotted against their gene expression level. A GAM smoothing curve is fit using all subjects. (JPG) [file pone.0205427.s004.jpg]
